# Erythrodontia in congenital erythropoietic porphyria

**DOI:** 10.4103/0973-029X.80022

**Published:** 2011

**Authors:** Rashmi Bhavasar, G Santoshkumar, B Rahul Prakash

**Affiliations:** *Department of Oral Pathology and Microbiology, Kamineni Institute of Dental Sciences, Narketpalli, Andhra Pradesh, India*; 1 *Department of Orthodontics, Kamineni Institute of Dental Sciences, Narketpalli, Andhra Pradesh, India*; 2 *Department of Pharmacology, Kamineni Institute of Dental Sciences, Narketpalli, Andhra Pradesh, India*

**Keywords:** Congenital erythropoietic porphyria, erythrodontia, Günther’s disease, porphyria

## Abstract

Congenital erythropoietic porphyria (CEP) is one of the rarest of porphyrias occurring worldwide. CEP is a very rare genetic autosomal recessive disease, with mutation in the gene that codifies uroporphyrinogen-III synthase, leading to porphyrin accumulation in many tissues, with marked skin photosensitivity, hemolytic anemia with splenomegaly and a decreased life expectancy. We report a case of Gü;nther’s disease in view of its rarity along with a description of this interesting condition. An 18-month-old female baby with clinical, hematological and biochemical profile of CEP was reported with marked skin photosensitivity over face and hands. She had erythrodontia with delayed eruption of teeth. When evaluating erythrodontia of uncertain cause, we advocate maintaining a high degree of awareness for porphyria, especially for CEP as it is the rarest among porphyria and is a life-threatening condition.

## INTRODUCTION

As befits a hematological class of disorders, the word “porphyria” derives from the Greek word porphuros which means red or purple.[1] Congenital erythropoietic porphyria (CEP) is also known as Günther’s Disease[2] was originally named hematoporphyria by Günther in 1911, and the first published case of porphyria was probably of this type.[2]

It is one of the rarest cutaneous porphyrias with approximately 150 cases reported till date.[3] CEP is a genetic autosomal recessive disease can occur from infancy, and has decreased life expectancy. The uroporphyrin (URO)-III s is one of the enzymes responsible for the synthesis of heme in erythrocytes. Physiologically, URO-III s converts hydroxymetilbilane into uroporphyrinogene-III. In URO-III s deficiency almost 85% of hydroxymetilbilane spontaneously condenses into isomer-I or biologically inactive URO-I, while 15% condenses into isomer-III (uroporphynogene-III).[4] URO I mainly accumulate in the bones, erythrocytes, skin and teeth accompanied by the excretion of large quantities of porphyrins in the urine and feces. Uroporphyrins and other metabolic deposits in the skin produce oxidative damage when exposure to light occurs.

In this case report, we focus on a case of CEP who was presented with erythrodontia and associated hemolytic anemia.

## CASE REPORT

An 18-month-old baby girl, product of a consanguineous marriage, second in birth order was presented to Kamineni Institute of Medical Sciences, Narketpally, Nalgonda, Andhra Pradesh, India. She had a history of blisters over face, hands and feet on exposure to sunlight since the 6^th^ day of birth. Her parents have noticed red discoloration of urine since 8^th^ day of birth.

The general physical examination revealed a baby girl with height and weight below the average for age.

**Figure 1:**
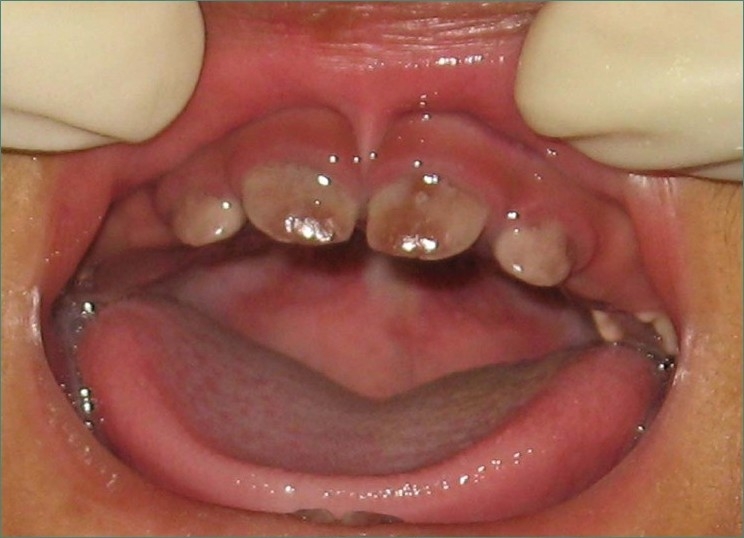
Photograph showing reddish brown discoloration of teeth

Intraoral examination revealed a reddish, brown opalescent discoloration of teeth in normal light (Erythrodontia), with hypertrophic gums [Figure 1]. No other lesions were seen in oral cavity.

**Figure 2:**
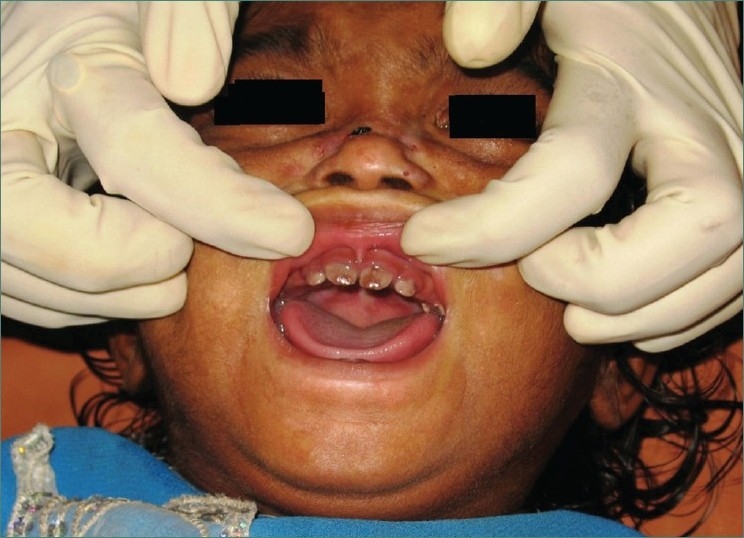
Photograph showing reddish brown discoloration of 51, 52, 61 and 62 and partially erupted 64

**Figure 3:**
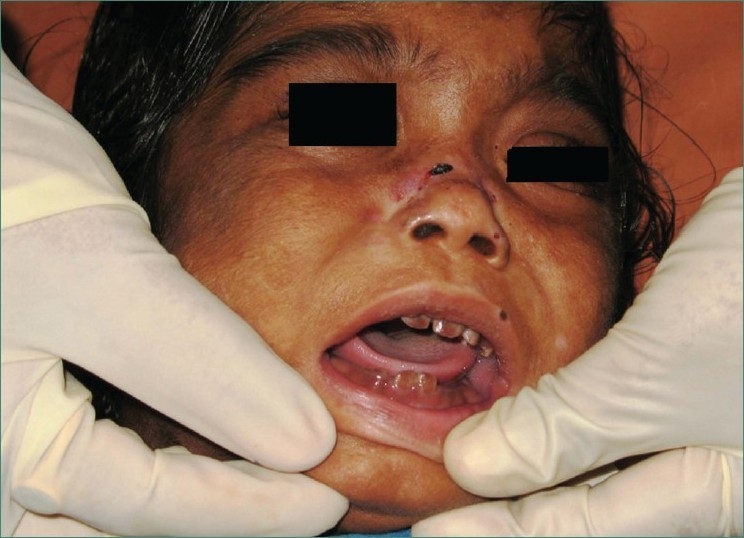
Photograph showing reddish brown discoloration of 51, 52, 61, 62, 71 and 81 and partially erupted 82

**Figure 4:**
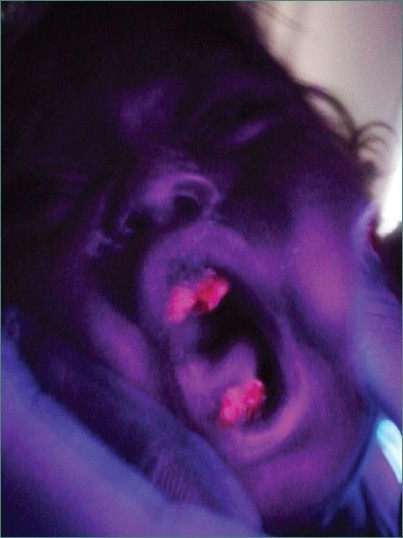
Photograph showing presence of bright red fluorescence of upper and lower central incisors under Wood’s lamp examination

**Figure 5:**
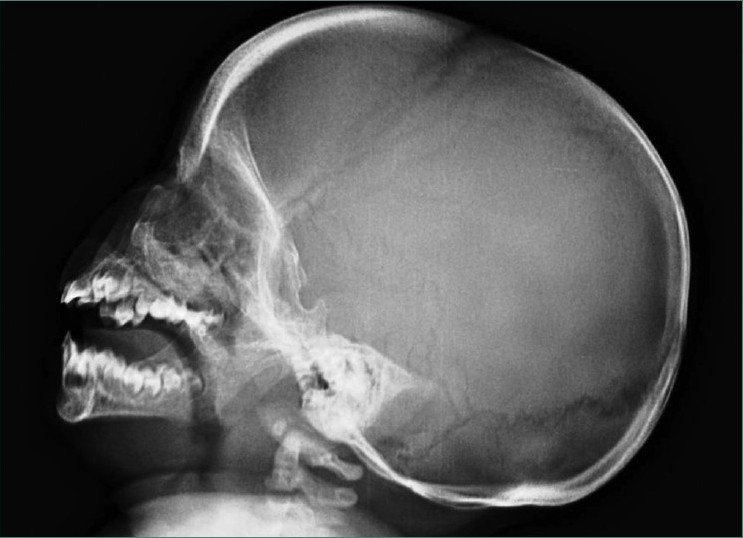
Lateral view of skull

Teeth present were 51, 52, 61, 62, 71 and 81 [Figures 2 and 3] and partially erupted teeth were 64 and 82 history of delayed eruption of teeth. Under Wood’s lamp there was a bright red fluorescence in the teeth [Figure 4]. Radiological examination revealed widening of diploic space in frontal and occipital areas but it may be considered normal for such a small age of patient. Teeth appears to have slightly delayed eruption and density of enamel and dentine nearly same [Figure 5].

**Figure 6:**
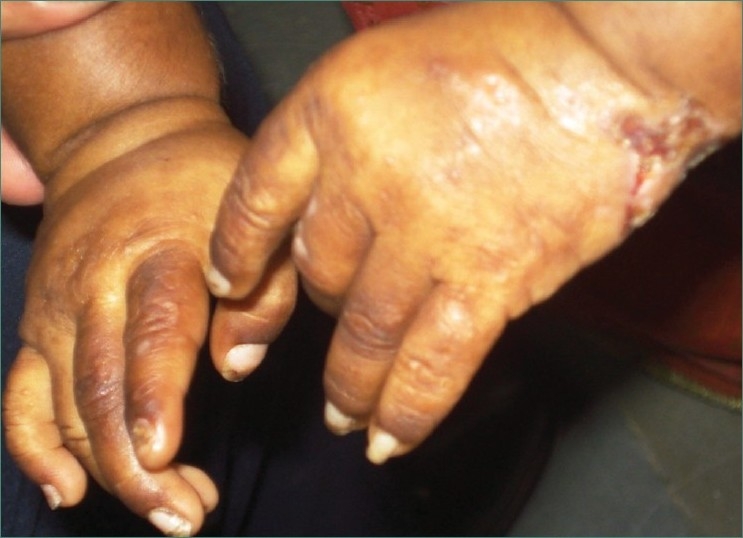
Photograph showing presence of lesions and scars on both hands of patient

**Figure 7:**
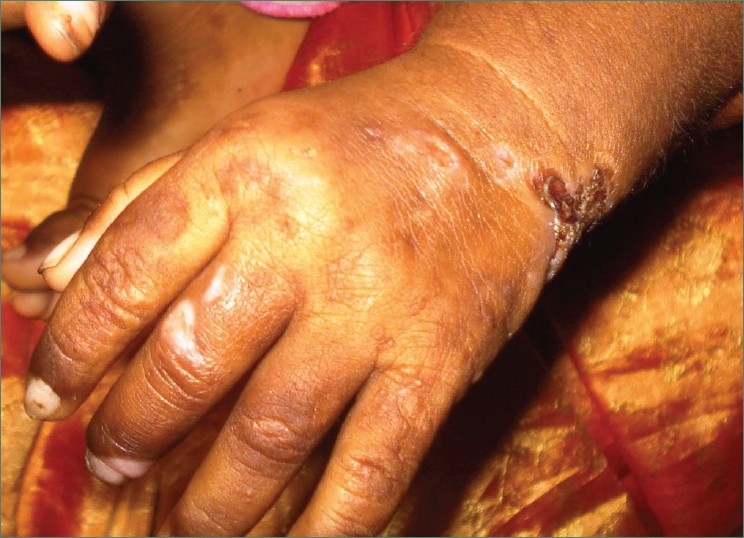
Photograph showing presence of lesions on fingers of left hand of patient

**Figure 8:**
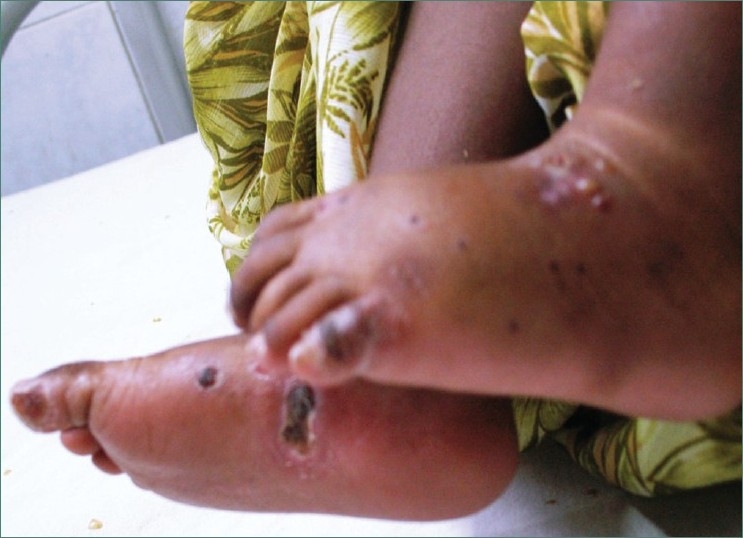
Photograph showing presence of multiple recurring lesions and scars on legs of patient along with pedal edema

On cutaneous examination hypertrichosis was present on face and back of patient along with blisters on face, nose, hands [Figures 6 and 7] and feet [Figure 8]. In addition to this milia, hyper and hypopigmentation and residual atrophic scars were also present on the face.

Abdominal examination revealed hepatosplenomegaly on ultrasonography. Central nervous system and respiratory systems were normal.

Routine laboratory investigations were as follows on the day of admission.

Hemoglobin – 3.7 gm%; TC – 11,000/cumm; DC – N 56, L 40, E 02, M 02, B 00 %.

**Figure 9:**
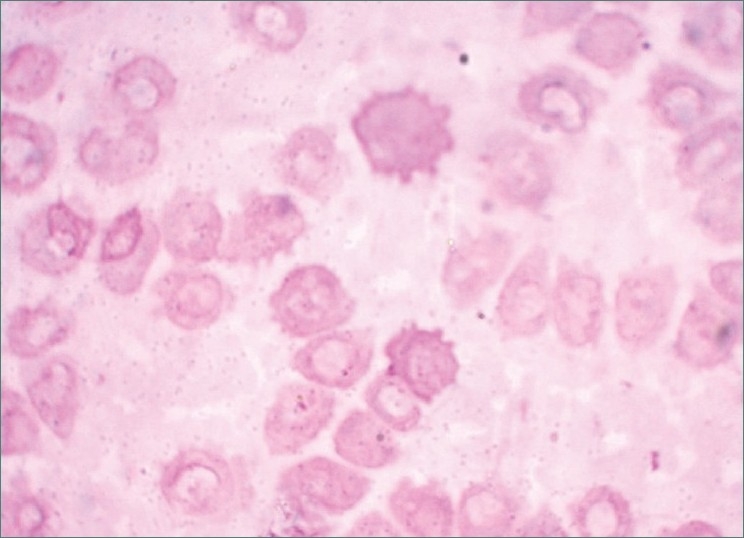
Photomicrograph showing presence of microcytic; hypo chromic RBCs with polychromasia of few cells. (100×)

**Figure 10:**
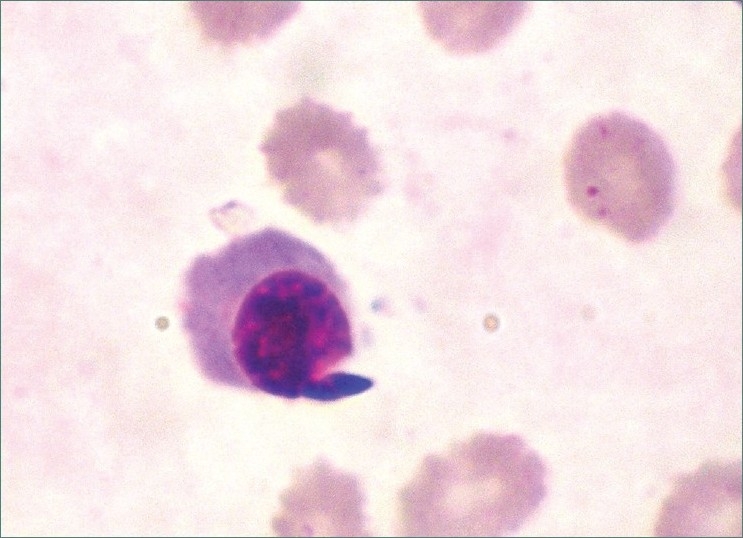
Photomicrograph showing presence of a normoblast. (100×)

Platelet reduced to 60,000/cumm and WBC was adequate on smear. Peripheral blood film examination revealed microcytic, hypochromic RBC with moderate anisopoikilocytosis, polychromasia of few cells [Figure 9] and presence of target cells. There was also the presence of a few normoblast [Figure 10]. Impression was suggestive of hemolytic anemia with thrombocytopenia. Liver function tests showed an elevated serum bilirubin, SGOT and raised alkaline phosphatase levels. Urine sample was negative for porphobilinogen.

Under Wood’s lamp there was a bright red fluorescence in the teeth, urine and blood. In our patient there were elevated levels of both uroporphyrins and coproporphyrins in the erythrocytes, higher levels of urinary porphyrin excretion were suggestive of CEP.

Erythropoietic protoporphyria (EP) was ruled out on the basis of biochemical methods as there was fluorescence in urine under Woods lamp. We tried to differentiate between CEP and hepatoerythropoietic porphyria (HEP) by separating out the porphyrins (uroporphyrins, coproporphyrins and protoporphyrins) in the erythrocytes by the method of Remington (as modified by Moore)[5] as in CEP, uroporphyrins and coproporphyrins will be raised and in HEP, Zn proto-porphyrins will be raised. In our patient, there were elevated levels of both uroporphyrins and coproporphyrins in the erythrocytes suggesting a diagnosis of CEP. Still HEP cannot be ruled out unless being confirmed by the absence of 7-carboxylate porphyrins in urine and isocoproporphyrins in stool by high pressure liquid chromatography (HPLC).[6]

HPLC was not possible in our patient because of cost considerations.

The patient was given blood transfusion to achieve sufficient levels of hemoglobin and also was asked to use topical sunscreens and β-carotene and is on regular follow-up.

## DISCUSSION

CEP has been observed to occur in countries around the world. Schultz is credited with the first clinical description of CEP.[7]

CEP is a rare genetic autosomal recessive disease that results from deficient activity of URO-III synthase, the fourth enzyme in the hemebiosynthetic pathway. The gene for the enzyme is found on chromosome 10q25.3 > q26.3. In CEP a variety of UROS mutations have been identified including large and small deletions and insertions and slicing defects.[4,8] The C73R mutation is the most frequent, and was found in ≈33%; of alleles. The URO synthase gene mutations occurring at p.Q249X and p.L237P in exon 10 were identified.[9] To date, more than 35 URO-synthase mutations have been identified in patients with CEP (Human Gene Mutation Database)[10] including 22 missense mutations whose mutant enzymes have varying amounts of residual activity. In fact, the presence and severity of clinical manifestations in CEP depend on the amount of residual URO-synthase activity and range from non-immune hydrops fetalis to a milder, later-onset form with only cutaneous photosensitivity and compensated hemolysis. Thus, phenotype-genotype correlations have been established for certain URO-synthase mutations.[11]

The clinical features include extreme photosensitivity either from birth or with a delayed onset leading to erythema, edema and blisters on the photo-exposed parts.[4]

In our patient, lesions healed over time with scarring, milia, hypo and hyperpigmentation. Repeated episodes of blistering result in mutilation of ears, nose and hands. Hypertrichosis is also present. As the porphyrins within the RBCs in the cutaneous microvasculature are exposed to light, hemolytic anemia can occur. This may be severe enough to induce marrow hyperplasia often with visible expansion of the maxillary bones in the face.[4] It may also lead to gallstones, splenomegaly, acro-osteolysis, osteopenia and compression fractures.[4]

There is passage of port wine-colored urine. The typical biochemical findings include elevated levels of uroporphyrin in urine with levels even upto 60 times than normal. Coproporphyrin I is also present in urine and excreted in large amounts in feces.[4]

The severe photosensitivity; red fluorescent urine and erythrodontia are usually diagnostic of EP. The close differential diagnoses are EP and porphyria cutanea tarda. In EP, hemolysis, erythrodontia and hirsutism never occur. Hepatic damage is seen and no change in color of urine occurs as protoporphyrin is excreted only in bile and feces and is present in RBCs.[4]

Porphyria cutanea tarda is generally late in onset. Photosensitivity is mild and liver damage is common. It may be associated with diabetes mellitus, systemic lupus erythematosus, uremia and certain immunological disorders. Characteristically uroporphyrin and coporphyrin are present in urine but RBCs are devoid of them.[8]

The present patient had severe photosensitivity, red-colored urine, hemolytic anemia but no gall stone was found in ultrasonography, pointing toward CEP.

For the differential diagnosis of Erythrodontia we can consider,

### Erythroblastosis fetalis

Teeth will have green, brown or blue hue by deposition of blood pigment in the enamel and dentin of the developing teeth. But here stain does not involve teeth or portions of teeth developing after cessation of hemolysis shortly after birth. Also ground sections of these teeth will be positive for bilirubin.[12]

Pink tooth of Mummery where internal resorption begins centrally within the tooth is apparently initiated in most cases by a peculiar inflammatory hyperplasia of pulp. But it is unusual for more than one tooth in any given patient to be affected by internal resorption, although cases of multiple tooth involvement have been recorded. But it would not involve both dentitions and all teeth at a time as seen in CEP.

As seen in Turner’s tooth, local infection or trauma can cause brownish discoloration of a single tooth but not involving all teeth as seen in CEP. In Fluorosis also mottled enamel frequently becomes stained an unsightly brown discoloration, but it will range from whitish spots to pitting discoloration.

Also enamel hypoplasia due to tetracycline staining will lead to discoloration of teeth but it will not involve both dentitions and all teeth at a time as seen in CEP.

Here we are considering tobacco stain, brown stain and orange stain for differential diagnosis but it is very clear that these are not intrinsic stains as those of porphyria. Treatment includes symptomatic measures in the form of sun protection,[13] β-carotene and also splenectomy for intractable hemolytic anemia. Transfusion of erythrocytes, intravenous hematin and oral-activated charcoal, bone marrow transplant (BMT) and gene therapy is also advocated. For severely affected patients BMT has been curative.[14,15] However, BMT requires at least a haploidentical match and is associated with a significant risk of morbidity and mortality.[13] Thus efforts have been directed to develop stem cell gene therapy for CEP.[16]

With the above-mentioned constellation of clinical, hematological and biochemical features, a final diagnosis of CEP with hemolytic anemia was entertained and is being reported in view of the rarity of this condition.

Characterisitic that dentists, should not neglect erythrodontia as it is a very rare and is one of the differentiating sign of CEP from that of other types of porphyria like porphyria cutanea tarda. A number of different types of porphyria may exhibit oral manifestations. These may be usually varied, but often are so characteristics that dentist should strongly suspect, if not actually confirm, the diagnosis of CEP. Thus dental manifestations are associated with a variety of skin diseases that can be genetic and an appreciation and understanding of dental signs can aid in the diagnosis and treatment of skin conditions.
